# The Assessment of the Supply of Calcium and Vitamin D in the Diet of Women Regularly Practicing Sport

**DOI:** 10.1155/2019/9214926

**Published:** 2019-11-04

**Authors:** Michał Wrzosek, Jakub Woźniak, Dorota Kozioł-Kaczorek, Dariusz Włodarek

**Affiliations:** ^1^Department of Dietetics, Faculty of Human Nutrition and Consumer Sciences, Warsaw University of Life Sciences (WULS–SGGW), Nowoursynowska 159 C, 02-776 Warsaw, Poland; ^2^Departments of Agricultural Economics and International Economic Relations, Faculty of Economic Sciences, Warsaw University of Life Sciences (WULS–SGGW), Nowoursynowska 159 C, 02-776 Warsaw, Poland

## Abstract

**Introduction:**

The appropriate intake of calcium and vitamin D in women's diet is significant for a proper maintenance of the skeletal system.

**Research Aim:**

The aim of the research was to assess the calcium and vitamin D supply in a diet among women regularly practicing sport.

**Methodology:**

The research was completed by 593 women at the age of 18–50 (median 25) who played sports regularly (at least 2 times a week). To assess the calcium and vitamin D intake, short Food Frequency Questionnaires for calcium and vitamin D (VIDEO-FFQ) were used. The examined group was provided with the questionnaires via social media. To assess intake levels, the authors applied the group-based cutoff point method (calcium norm was EAR 800 mg/day; vitamin D norm was AI 15 *μ*g/day).

**Results:**

The median of calcium and vitamin D intake in a diet was 502 mg/day and 5.2 *μ*g/day, respectively (Q25 and Q75 for calcium was 387 mg/day and 627 mg/day, respectively, and for vitamin D was 3.4 *μ*g/day and 8.2 *μ*g/day, respectively). In relation to the EAR norm for calcium and AI norm for vitamin D, 92.0% of the examined participants in a group demonstrated lower than recommended calcium intake levels and 97.3% showed lower than recommended vitamin D intake levels. Calcium and vitamin D supplementation was used by 13.1% (in this subgroup, 11.5% of the examined group members did not need it) and 56.8% of the examined women (in this subgroup, 2.4% of the examined group did not need it), respectively. After including the calcium and vitamin D intake, the supply median for the whole group was 535 mg/day and 28.8 *μ*g/day, respectively (Q25 and Q75 for calcium was 402 mg/day and 671 mg/day, and for vitamin D was 6.3 *µ*g/day and 55.7 *μ*g/day, respectively); 87.5% of the examined participants did not meet the EAR norms for calcium and 42.0% did not meet the AI norm for vitamin D. Among the women supplementing calcium, 58.9% did not reach the reference intake value; however, all women supplementing vitamin D fulfilled the expected nutritional need.

**Conclusions:**

It is important to educate women about the necessity to provide the body with proper calcium and vitamin D intake levels in a diet in order to avoid health problems resulting from the deficit of the nutrients.

## 1. Introduction

The dynamic of bone tissue metabolism undergoes many changes with age. There is a constant increase of bone mineral density (BMD) when one is at a young age, and it lasts until the third decade of life when the osteoblastic processes are inhibited. Although the structural modelling of bones is the most optimal during puberty, this process continues, albeit to a smaller extent, when the body reaches maturity. Over the age of 30, the bone-forming processes are recurrent and are strictly dependent on the external factors, including increased physical activity. The peak bone mass gained between the age of 25 and 35 decreases over time [1, 2]. It is estimated that the loss of bone mineral density proceeds at a pace of 1% per year and escalates amongst postmenopausal women [3]. When intensified, osteoclastic processes increase the risk of osteoporosis in later years. The frequency of its occurrence among women is almost fourfold higher when compared to men [4]. It shows that it is women rather than men who require special therapeutic observations in terms of preventing osteoporosis. Among the factors influencing bone mineral density one may find eating habits and body nutrition, genetic and hormonal factors, physical activity, smoking, and drinking alcohol [3, 5].

Physical activity, which is the external factor, stimulates a constant rebuilding and supercompensation of the skeletal system [6]. Despite influencing the skeletal system, regular physical activity increases body mass and muscle power which is related to mobility and overall health condition [7]. Regular trainings and participation in competitions influence the increase of endurance of the skeletal system at a young age. This process varies between different physical activities. Resistance exercises and overload activities (running, football, and rugby) have the greatest impact on BMD, whereas the lack of physical activity has a neutral or negative impact on it [8]. In the research based on a group of 30 young rugby players (aged 21.4 ± 1.9), scans showed that their BMD levels were significantly higher than those of their peers who did not do any sports [9]. The correlation was also confirmed in another research based on women doing sports [10]. There is a very strong correlation between the obtained bone mass levels during puberty and bone resilience in later years [11–14]. This thesis seems to be confirmed by the retrospective examinations of athletes which indicated a positive impact of physical activity on developing bone mass which, however, may decrease in the absence of physical activity [15–19].

Eating habits also have an impact on the levels of bone mass in women doing sports. Current studies pay particular attention to calcium and vitamin D intake in a diet [20–22]. Apart from the intake of the nutrients, BMD is also influenced by calcium bioavailability and vitamin D endogenous synthesis in human skin.

As one of the mineral components, calcium is a basic bone-building nutrient. Its proper intake indirectly impacts the rate of bone growth at a young age and the maintenance of the optimal mineralization of the skeletal system in later years [5, 23, 24]. According to the Polish Recommended Dietary Allowance (RDA), the recommended daily value for calcium for women between 19 and 65 is 1000 mg [25], 75% of which is, as indicated by observatory research, fulfilled by the consumption of dairy products [26].

The proper calcium intake in a diet, along with the proper physical activity level, determines the dominance of osteoblastic processes over osteoclastic processes. Furthermore, insufficient intake levels leading to negative calcium balance result in bone decalcification and a reduction of bone mineral density in the long term [27]. Various studies showed that the calcium intake in a diet of people doing sports is not sufficient [28–30]. The research conducted on 41 girls doing sports showed that the average calcium intake in a diet was 855 mg, and only 11.6% of the participants obtained the recommended daily value for the mineral component [31]. Barrack et al. in turn, observed in their research that 85% of the examined runners did not fulfil a daily norm of calcium intake [32].

Vitamin D belongs to a group of fat-soluble vitamins which have a broad biological impact. In addition to its impact on calcium-phosphate metabolism, vitamin D also influences the immune system, steroid hormones balance, and athletes' manual effort capacities [33, 34]. Due to low vitamin D intake in a diet, the athletes' main source of the vitamin is supplementation and endogenous synthesis in skin [35]. Under the influence of UV-B radiation, a derivative of cholesterol in human skin cells undergoes a transformation: 7-dehydrocholesterol to cholecalciferol which is firstly transformed in the liver and then in the kidneys into an active form of cholecalciferol, that is, 1,25-hydroxyvitamin D (1.25 (OH) D) [36]. The level of body saturation with vitamin D is established on the basis of concentration of its 25(OH)D metabolite in blood. Values under 30 ng/ml are deficient, whereas the optimal level of vitamin D for athletes is 40–60 ng/ml [36]. It appears that the risk of vitamin D deficiency among athletes may be related to the kind of practiced sport and the latitude where the trainings take place. Female runners training outside are less likely to demonstrate vitamin D deficiency, which is related to regular skin synthesis [37]. The research conducted on 103 athletes living in the south of the United States of America showed that only 32% of them had vitamin D deficiency (lower than 30 ng/ml 0.25 (OH) D in blood serum) [38]. In regions where athletes are less exposed to sunlight, skin synthesis of vitamin D does not take place optimally, which should encourage the supplementation of this vitamin. Especially that the deficiency of cholecalciferol is correlated with lower bone mass and a higher risk of bone fractures in the population [39]. In women, adequate supply of vitamin D is of great importance at the age of 15 to 30 years, when they reach peak bone mass, maximum bone strength, and maximum density [40]. This statement is confirmed by the cross-sectional study, in which 400 patients of the university hospital participated. This study showed a positive correlation between BMD and the concentration of vitamin D metabolite (0.25 (OH) D) in the serum of the subjects [41]. A similar observation was reported by Roy et al. in a study involving 78 women in which the concentration of vitamin D was positively correlated with the density of the femur and spine in the lumbosacral segment [42].

The research aimed to assess the supply of calcium and vitamin D in a diet and supplementation among women regularly practicing sport.

## 2. Methods

The research was completed by 593 women aged 18–50 (median 25) regularly practicing sport. The minimum required training frequency was 2 workouts a week, while the most common training frequency was 3-4 workouts a week. The most commonly performed female activity was strength training. Only 19 women were professional athletes, and the remaining 574 women practiced amateur sport. The median of body mass among group participants was 63 kg (min. 46 and max. 95 kg). The BMI of most of the women was within the proper limits, 18.5–25 (in the examined group BMI for 25 quintiles was 20.9 and for 74 quintiles was 24.3). To assess the vitamin D intake level, Vitamin D the Estimation Only Food Frequency Questionnaire (VIDEO-FFQ) was used [43]. This questionnaire has been validated for the Polish population and developed based on typical European food products or groups of food products. The VIDEO-FFQ obtained a value of less than 5.0% for the Bland–Altman plot, which can be interpreted as an important and repeatable tool to assess vitamin D intake in women living in Poland [43]. In addition, the questionnaire was successfully adapted to the needs of the region of Croatia where it was also validated, which enabled its use also in another European country [44]. Calcium intake, in turn, was assessed by means of a validated questionnaire assessing calcium obtained from dairy products and other food product intake [45, 46]. Calcium consumption was assessed by summing the intake of calcium obtained from dairy products and 250 mg of calcium being an estimated intake of this mineral component from other products [46]. Moreover, while estimating the calcium and vitamin D intake, their supplementation was taken into consideration. The examined group was provided with the questionnaires via social media. The questionnaires were approved by the local Ethics and Scientific Research on Humans Commission of Faculty of Human Nutrition and Consumer Sciences–SGGW (Warsaw University of Life Sciences) (approval number: 17P/2018, July 5, 2018). The results of calcium intake referred to EAR, and the results of vitamin D intake for the population of Poland referred to AI [47]. To assess the intake of the discussed nutritional components, the cutoff point method was used. To verify data distribution, the Shapiro–Wilk test was used. The correlation between vitamin D and calcium intake was checked by means of Spearman's test. The study's defined significance level was set to *p* ≤ 0.05. Participation in the study has been voluntary. Data have been collected via the Instagram social networking site. Respondents were asked to fill in a questionnaire on a special website.

## 3. Results

The median of calcium and vitamin D intake in a diet was 502 mg/day and 209 IU/day, respectively (Table 1).

Among the products that have the greatest impact on calcium intake, the authors included milk, yoghurt, hard cheese, and cottage cheese, respectively. The products that provide the body with the highest vitamin D intake levels were mostly dairy products, and then eggs, fish, and their preserves. Calcium and vitamin D intake obtained from particular products is presented in Tables 2 and 3.

According to EAR and AI norms, only 8% of the examined participants fulfilled the need for calcium and 2.7% of the examined group fulfilled the need of vitamin D, taking account of dietary sources alone. A significant source of vitamin D in the examined participants was supplementation. However, as regards the intake of calcium, the use of supplementation was much rarer amongst the participants. Only 13.1% of the women supplemented calcium, and 56.8% of the women supplemented vitamin D. The most frequently supplemented quantity of vitamin D was 1000 IU, and the highest daily intake of vitamin D was 8000 IU (as in the case of one of the research participants). Only 41.1% of women supplementing calcium reached the intake level consistent with the EAR norm, and as many as 58.9% did not supplement adequate amounts of nutrients. In this group, 11.5% of women did not need supplementation. As regards vitamin D, only 2.4% of women need not have supplemented vitamin D. Moreover, the dose fulfilled the needs of the women in 100% (according to the AI norm).

Having included calcium and vitamin D supplementation, the intake median in the group was 535 mg/day and 1152 IU/day, respectively (Q25 and Q75 for calcium was 402 mg/day and 671 mg/day, respectively, and for vitamin D was 252 IU/day and 2226 IU/day, respectively) (Table 3), and the EAR recommended daily value for calcium was not obtained by 87.5% of the examined athletes, whereas the recommended AI daily value for vitamin D was not obtained by 42.0% of the examined group. In other words, only 12.5% of women have achieved the EAR standard for calcium and 58% of women have completed the AI standard for vitamin D.

Calcium intake among the participants was highly correlated with the consumption of such products as milk, yoghurt, hard cheese, and cottage cheese, whereas vitamin D intake was highly correlated with dairy products and, to a lesser extent, fish, eggs, and meat products (Table 4).

## 4. Discussion

Insufficient calcium and vitamin D intake in a diet of physically active people is a regular phenomenon [28, 29]. The research conducted on 21 young women doing sports showed that the average calcium intake in a diet was 855 mg and only 11.6% of the participants obtained the recommended daily value for the mineral component [31]. In another research, only 15% of female runners fulfilled the calcium norm. Slightly different results were obtained by Eksterowicz and Napierała [48]. They showed in their research, which focused on analysing vitamins and mineral components intake in a diet conducted during a sport camp, that all the female participants fulfilled 100% of the calcium daily norm intake. It is worth noticing that the women's diet was planned by the sports centre, and they ate according to the planned diet. Even though the publication under discussion does not prove that the calcium intake in a diet is diverse, depending on the examined group, it shows that the diet of physically active women may provide the body with the appropriate calcium intake level. As underlined by the authors, the main sources of calcium during the sports camp were dairy products, which were included in the women's diet in the amount of 1200 g/day (including 300 g of milk). Unfortunately, the reduction of dairy products consumption and the lack of their well-balanced replacement in a diet of physically active people are the main factors leading to calcium deficiency. In our research and the research of Kosendiak et al. [49], positive correlation between low dairy products intake and Ca deficiency in a diet was observed. Undoubtedly, a reasonable replacement of the discussed products with plant-based ones, supplementation, and mineral water is possible and applied by some athletes. However, it is noteworthy that it requires a lot of knowledge or cooperation with a sports dietician.

Insufficient vitamin D intake is a problem that is often observed among athletes [37, 38]. In this research, only 2.7% of the examined women obtained the recommended daily value for vitamin D in a diet. Certainly, it relates to an insufficient intake of products including the vitamin, such as oily sea fish. Hence, for the practical purposes, vitamin D supplementation should be recommended, and, if possible, in the summertime, one should promote outdoor training. Nearly 57% of the examined women supplemented vitamin D. Similar results were obtained by Skop-Lewandowska et al. [50] since in their research the popularity of vitamin D supplementation among women was nearly 49%. In another study by Duch, which included research based on 128 professional athletes (including 48 women), the vast majority of the group participants (70% of the participants) demonstrated a considerable vitamin D deficit.

After a 12-month-long supplementary record including 2200 UI daily vitamin D intake, the rebalance of deficiency in 80% of the athletes was noticed [51]. It is worth noting that the deficiency of vitamin D does not affect only physically active people. Vitamin D deficiency affects the entire European society. The analysis of the research, which covered 55,844 people living in European countries, showed the concentration of vitamin D metabolite (25 (OH) D) below 30 nmol/l in 13% of people. This result means a deficiency in the bodies of subjects [52]. Vitamin D shows a positive impact on mineral balance and athlete's exercise capacity. According to AIS (Australian Institute of Sports), supplementation of vitamin D which aims to rebalance deficiencies is justified, and supplements including vitamin D are recommended (Group A) [53]. Despite the lack of the official guidelines concerning the amount of supplementation among the athletes, the recommendations for physically active people may be based on a recommendation for the population as a whole. They confirm that the recommended vitamin D dose for the adults should be from 800 to 2000 UI per day [54], whereas one should not take more than the 4000 UI dose without consulting the doctor [55]. Apart from vitamin D intake in a diet, together with pharmaceutical preparations, the athletes should be regularly exposed to the sun. However, it is very important to balance the benefits and drawbacks of sunlight exposure, which at least during the summertime satisfies the need for vitamin D intake although it increases the risk of skin cancer.

It should be noted that the limitation of the study was the assessment of the supply of vitamin D along with diet and supplementation. The concentration of vitamin D metabolite in the blood was not evaluated, which would be the best method to assess the saturation of this vitamin in the blood of the examined group.

## 5. Conclusion

To sum up, the results of the research allow us to draw the following conclusions:

1. Almost all of the examined women did not provide the body with the satisfying calcium and vitamin D intake in a diet
2. Only a part of the participants supplemented calcium and vitamin D, which helped some of them to obtain the recommended daily value
3. Vitamin D supplementation is much more popular than calcium supplementation, which resulted in more frequent Ca deficiency in the examined group
4. The dairy products were the most significant source of calcium and vitamin D in a diet
5. It is necessary to educate women about the importance of providing the body with the sufficient calcium and vitamin D intake in a diet in order to avoid health consequences resulting from the deficiency of the nutrients

## Figures and Tables

**Table 1:** Intake of calcium (mg) and vitamin D (IU) in a diet, including supplementation.

	Median	Q25	Q75	Minimum-maximum
Ca intake in a diet	502.8	387.1	627.1	264.3–1195.7
Ca intake in a diet including supplementation	535	402.1	670.7	267.1–2715.7
Vit. D intake in a diet	209	136.6	326.51	0–1036.4
Vit. D intake in a diet including supplementation	1152	252.3	2226	0–8739.7

**Table 2:** The examined group's Ca intake (mg/day) obtained from food products.

Products	Median	Q25	Q75	Minimum-maximum
Milk	68.5	34.2	102.85	0–240
Yoghurt	48.5	0	97.1	0–388.5
Hard cheese	21.4	0	85.7	0–214.2
Cottage cheese	28.5	0	57.1	0–285.7
Nutrient-enriched drinks	0	0	0	0–137.1
Nutrient-enriched products	0	0	17.1	0–154.2

**Table 3:** The examined group's vitamin D intake (IU/day) obtained from food products.

Products	Median	Q25	Q75	Maximum
Fish and their preserves	30.4	4	63.6	238.1
Dairy products	58.5	3.88	167.1	532.8
Eggs	48.5	24.2	72.8	170
Meat and its preserves	29.1	17.6	43.7	129.6
Butter and other fats	1.2	0	4.8	124
Pasta	0	0	2.8	14.3
Ice cream	0	0	0.4	3.2

**Table 4:** Correlation between calcium and vitamin D intake and consumption of particular products (*p* ≤ 0.05).

	Correlation coefficient
*Calcium*	
Yoghurt	0.73
Milk	0.66
Hard cheese	0.51
Cottage cheese	0.48
Nutrient-enriched products	0.42
Nutrient-enriched drinks	0.27
Vitamin D	
Dairy products	0.81
Fish and their preserves	0.38
Eggs	0.33
Meat and its preserves	0.27
Butter	0.25
Ice cream	0.13
Pasta	0.12

## Data Availability

The research article data used to support the findings of this study are available from the corresponding author upon request.

## References

[1] Frost H. M. (2000). Leczenie osteoporozy. Quo vadis?. *Medicina Sportiva Practica*.

[2] Smith R., Harrison J., Cooper C. (2000). *Osteoporoza*.

[3] Włodarek D. (2009). Znaczenie diety w zapobieganiu osteoporozie. *Endocrinology, Obesity and Metabolic Disorders*.

[4] Nguyen H. T., von Schoultz B., Nguyen T. V. (2015). Sex hormone levels as determinants of bone mineral density and osteoporosis in Vietnamese women and men. *Journal of Bone and Mineral Metabolism*.

[5] Heaney R. P. (2000). Calcium, dairy products and osteoporosis. *Journal of the American College of Nutrition*.

[6] Bandurski J. (2005). Choroby metaboliczne kości.

[7] Hara S., Yanagi H., Amagai H., Endoh K., Tsuchiya S., Tomura S. (2001). Effect of physical activity during teenage years, based on type of sport and duration of exercise, on bone mineral density of young, premenopausal Japanese women. *Calcified Tissue International*.

[8] Segev D., Hellesrstein D., Dunsky A. (2018). Physical acitivity-does it really increase bone density in postmenopausal women? A review of articles published between 2001–2016.

[9] Egan E., Reilly T., Giacomoni M., Redmond L., Turner C. (2006). Bone mineral density among female sports participants. *Bone*.

[10] Alfredson H., Nordström P., Lorentzon R. (1996). Total and regional bone mass in female soccer players. *Calcified Tissue International*.

[11] Goulding A., Jones I. E., Taylor R. W., Williams S. M., Manning P. J. (2001). Bone mineral density and body composition in boys with distal forearm fractures: a dual-energy X-ray absorptiometry study. *The Journal of Pediatrics*.

[12] Budek A. Z., Mark T., Michaelsen K. F., Mølgaard C. (2010). Tracking of size-adjusted bone mineral content and bone area in boys and girls from 10 to 17 years of age. *Osteoporosis International*.

[13] Cooper C., Westlake S., Harvey N., Javaid K., Dennison E., Hanson M. (2006). Review: developmental origins of osteoporotic fracture. *Osteoporosis International*.

[14] Hernandez C. J., Beaupr G. S., Carter D. R. (2003). A theoretical analysis of the relative influences of peak BMD, age-related bone loss and menopause on the development of osteoporosis. *Osteoporosis International*.

[15] Bass S., Pearce G., Bradney M. (1998). Exercise before puberty may confer residual benefits in bone density in adulthood: studies in active prepubertal and retired female gymnasts. *Journal of Bone and Mineral Research*.

[16] Khan K. M., Bennell K. L., Hopper J. L. (1998). Self-reported ballet classes undertaken at age 10–12 years and hip bone mineral density in later life. *Osteoporosis International*.

[17] Kontulainen S., Kannus P., Haapasalo H. (1999). Changes in bone mineral content with decreased training in competitive young adult tennis players and controls: a prospective 4-yr follow-up. *Medicine & Science in Sports & Exercise*.

[18] Michael B. A., Lane N. E., Bjorkengren A., Bloch D. A., Fries J. F. (1992). Impact of running on lumbar bone density: a 5-year longitudinal study. *The Journal of Rheumatology*.

[19] Vuori I., Heinonen A., Sievanen H., Kannus P., Pasanen M., Oja P. (1994). Effects of a unilateral strength training and detraining on bone mineral density and content in young women: a study of mechanical loading and deloading on human bones. *Calcified Tissue International*.

[20] Maugham R. J., King D. S., Lea T. (2004). Dietary supplements. *Journal of Sports Sciences*.

[21] New S. A. (2000). Wpływ żywienia na stan kości ze szczególnym uwzględnieniem wapnia i fosforu. współczesne poglądy w nauce. żywność, żywienie. *Prawo a Zdrowie*.

[22] Szkop J. (2001). Czynniki żywieniowe a metabolizm tkanki kostnej. *Żyw Człow Metab*.

[23] Flynn A. (2003). The role of dietary calcium in bone health. *Proceedings of the Nutrition Society*.

[24] Bonjour J.-P., Chevalley T., Ammann P., Slosman D., Rizzoli R. (2001). Gain in bone mineral mass in prepubertal girls 3–5 years after discontinuation of calcium supplementation: a follow-up study. *The Lancet*.

[25] Jarosz M., Bułhak-Jachymczuk B. (2017). *Normy Żywienia Człowieka. Podstawy Prewencji Otyłości I Chorób Niezakaźnych*.

[26] Brzozowska A., Składniki Mineralne W., Gawęcki J., Hryniewiecki L. (1998). *Żywienie Człowieka. Podstawy Nauki O Żywieniu*.

[27] Lorenc R. S., Karczmarewicz E. (2001). Znaczenie wapnia i witaminy D w optymalizacji masy kostnej oraz zapobieganiu i leczeniu osteoporozy u dzieci. *Ped. Współ., Gastroenterologia, Hepa- Tologia i Żywienie Dziecka*.

[28] Beshgetoor D., Nichols J. F., Rego I. (2000). Effect of training mode and calcium intake on bone mineral density in female master cyclists, runners, and non-athletes. *International Journal of Sport Nutrition and Exercise Metabolism*.

[29] Raczyńska B. (2001). Zaburzenia żywieniowe u zawodniczek. *Sport Wyczynowy*.

[30] Szczepańska B., Malczewska J. (2003). Zawartooeć energii i wybranych składników mineralnych w całodziennych racjach pokarmowych stosowanych w żywieniu polskich sportowców. *Żyw Człow Metab*.

[31] Szczepańska B., Malczewska-Lenczowska J., Wajszczyk B. (2011). Ocena spożycia witamin i składników mineralnych przez dziewczęta z warszawskiego gimnazjum sportowego. *Porbl Hig Epidemiol*.

[32] Barrack M. T., Van Loan M. D., Rauh M. J., Nichols J. F. (2010). Physiologic and behavioral indicators of energy deficiency in female adolescent runners with elevated bone turnover. *The American Journal of Clinical Nutrition*.

[33] Nickols-Richardson S. M., Beiseigel J. M., Gwazdauskas F. C. (2006). Eating restraint is negatively associated with biomarkers of bone turnover but not measurements of bone mineral density in young women. *Journal of the American Dietetic Association*.

[34] Nattiv A., Loucks A. B., Manore M. M. (2007). American college of sports medicine position stand. The female athlete triad. *Medicine and Science in Sports and Exercise*.

[35] Close G. L., Hamilton D. L., Philp A., Burke L. M., Morton J. P. (2016). New strategies in sport nutrition to increase exercise performance. *Free Radical Biology and Medicine*.

[36] Bouillon R., Carmeliet G., Verlinden L. (2008). Vitamin D and human health: lessons from vitamin D receptor null mice. *Endocrine Reviews*.

[37] Wentz L. M., Liu P.-Y., Ilich J. Z., Haymes E. M. (2016). Female distance runners training in southeastern United States have adequate vitamin D status. *International Journal of Sport Nutrition and Exercise Metabolism*.

[38] Hildebrand R. A., Miller B., Warren A., Hildebrand D., Smith B. J. (2016). Compromised vitamin D status negatively affects muscular strength and power of collegiate athletes. *International Journal of Sport Nutrition and Exercise Metabolism*.

[39] Cranney A., Horsley T., O’Donnell S. (2007). Effectiveness and safety of vitamin D in relation to bone health. *Evidence Report/Technology Assessment*.

[40] Benjamin R. M. (2010). Bone health: preventing osteoporosis. *Journal of the American Dietetic Association*.

[41] Sadat-Ali M., Al Elq A. H., Al-Turki H. A., Al-Mulhim F. A., Al-Ali A. K. (2011). Influence of vitamin D levels on bone mineral density and osteoporosis. *Annals of Saudi Medicine*.

[42] Roy D. K., Berry J. L., Pye S. R. (2007). Vitamin D status and bone mass in UK South Asian women. *Bone*.

[43] Głąbska D., Guzek D., Sidor P., Włodarek D. (2016). Vitamin D dietary intake questionnaire validation conducted among young polish women. *Nutrients*.

[44] Głąbska D., Uroić V., Guzek D. (2018). The possibility of applying the vitamin D brief food frequency questionnaire as a tool for a country with no vitamin D data in food composition tables. *Nutrients*.

[45] Włodarek D., Głąbska D., Kołota A. (2012). Calcium intake and osteoporosis: the influnce of calcium intake from dairy products on hip bone mineral density and fracture incidence—a population-based study in women over 55 years of age. *Public Health Nutrition*.

[46] Włodarek D., Głąbska D., Lange E. (2014). The effect of dairy products choice on calcium dietary intake in female university students of nutritional faculty. *Roczniki Państwowego Zakładu Higieny*.

[47] Jarosz M. (2012). *Normy Żywienia dla Populacji Polskiej—Nowelizacja*.

[48] Eksterowicz J., Napierała M. (2008). Ocena sposobu żywienia studentek z kierunku wychowania fizycznego podczas letniego obozu sportowego. *Roczniki Państwowego Zakładu Higieny*.

[49] Kosendiak A., Bronkowska M., Felińczak A., Biernat J. (2016). Ocena spożycia wybranych produktów mlecznych jako źródeł wapnia przez osoby przygotowujące się do maratonu. *Bromatologia i Chemia Toksykologiczna*.

[50] Skop-Lewandowska A., Małek A., Gmur M., Kolarzyk E. (2013). Sposób żywienia oraz popularność stosowania suplementów diety i odżywek wśród młodych osób uczęszczających do klubów fitness. *Problemy Higieny i Epidemiologii*.

[51] Backx E. M. P., Tieland M., Maase K. (2016). The impact of 1-year vitamin D supplementation on vitamin D status in athletes: a dose-response study. *European Journal of Clinical Nutrition*.

[52] Cashamn K. D., Dowling K. G., Skrabova Z. (2016). Vitamin D deficiency in Europe: pandemic?. *American Society for Clinical Nutrition*.

[53] Burke L., Deakin V. (2015). *Clinical Sport Nutrition*.

[54] Płudowski P., Karczmarewicz E., Bayer M. (2013). Practical guidelines for the supplementation of vitamin D and the treatment of deficits in Central Europe—recommended vitamin D intakes in the general population and groups at risk of vitamin D deficiency. *Endokrynologia Polska*.

[55] European Food Safety Authority (2012). Scientific opinion on the tolerable upper intake level of vitamin D. EFSA panel on dietetic products, nutrition and allergies (NDA). *EFSA Journal*.

